# Ethyl 1-*sec*-butyl-2-(4-methoxy­phen­yl)-1*H*-benzimidazole-5-carboxyl­ate

**DOI:** 10.1107/S1600536810007634

**Published:** 2010-03-17

**Authors:** Natarajan Arumugam, Aisyah Saad Abdul Rahim, Hasnah Osman, Madhukar Hemamalini, Hoong-Kun Fun

**Affiliations:** a School of Pharmaceutical Sciences, Universiti Sains Malaysia, 11800 USM, Penang, Malaysia; b School of Chemical Sciences, Universiti Sains Malaysia, 11800 USM, Penang, Malaysia; c X-ray Crystallography Unit, School of Physics, Universiti Sains Malaysia, 11800 USM, Penang, Malaysia

## Abstract

In the title mol­ecule, C_21_H_24_N_2_O_3_, the dihedral angle between the benzene and imidazole rings is 66.33 (13)°. The imidazole ring is essentially planar, with a maximum deviation of 0.004 (2) Å. In the crystal structure, mol­ecules are connected by weak C—H⋯O hydrogen bonds, forming chains along the *b* axis

## Related literature

For the benzimidazole nucleus as a key building block for compounds showing biologically activity, see: Tanious *et al.* (2004 ▶). For the therapeutic properties of benzimidazole derivatives, see: Kohara *et al.* (1996 ▶); Mader *et al.* (2008 ▶). For 2-substituted-phen­ylbenzimidazoles with biological activity, see: Coburn *et al.* (1987 ▶); Roth *et al.* (1997 ▶).
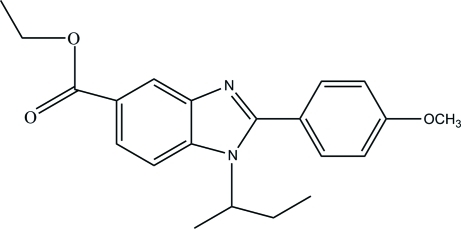


## Experimental

### 

#### Crystal data

C_21_H_24_N_2_O_3_
*M* *_r_* = 352.42
Monoclinic, 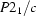

*a* = 10.5815 (3) Å
*b* = 12.1079 (3) Å
*c* = 15.1050 (3) Å
β = 93.678 (2)°
*V* = 1931.26 (8) Å^3^
*Z* = 4
Mo *K*α radiation
μ = 0.08 mm^−1^
*T* = 296 K
0.40 × 0.31 × 0.07 mm

#### Data collection

Bruker SMART APEXII CCD area-detector diffractometer
Absorption correction: multi-scan (*SADABS*; Bruker, 2009 ▶) *T*_min_ = 0.968, *T*_max_ = 0.994
19216 measured reflections
4437 independent reflections
2266 reflections with *I* > 2s(*I*)
*R*_int_ = 0.065

#### Refinement

*R*[*F* ^2^ > 2σ(*F* ^2^)] = 0.066
*wR*(*F* ^2^) = 0.150
*S* = 1.04
4437 reflections
235 parameters
H-atom parameters constrained
Δρ_max_ = 0.13 e Å^−3^
Δρ_min_ = −0.18 e Å^−3^

### 

Data collection: *APEX2* (Bruker, 2009 ▶); cell refinement: *SAINT* (Bruker, 2009 ▶); data reduction: *SAINT*; program(s) used to solve structure: *SHELXTL* (Sheldrick, 2008 ▶); program(s) used to refine structure: *SHELXTL*; molecular graphics: *SHELXTL*; software used to prepare material for publication: *SHELXTL* and *PLATON* (Spek, 2009 ▶).

## Supplementary Material

Crystal structure: contains datablocks global, I. DOI: 10.1107/S1600536810007634/tk2635sup1.cif

Structure factors: contains datablocks I. DOI: 10.1107/S1600536810007634/tk2635Isup2.hkl

Additional supplementary materials:  crystallographic information; 3D view; checkCIF report

## Figures and Tables

**Table 1:** Hydrogen-bond geometry (Å, °)

*D*—H⋯*A*	*D*—H	H⋯*A*	*D*⋯*A*	*D*—H⋯*A*
C21—H21*C*⋯O1^i^	0.96	2.47	3.389 (4)	161

Symmetry code: (i) 
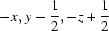
.

**Fig. 1.**
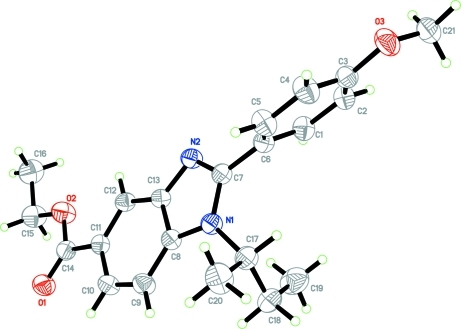
The title molecule, showing 50% probability displacement ellipsoids and the atom-numbering scheme.

**Fig. 2.**
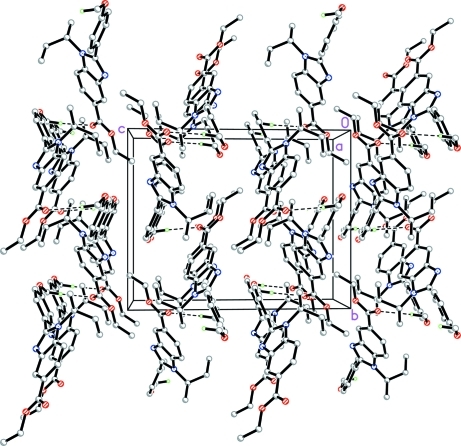
A view of the crystal packing of the title compound, showing C–H···O interactions as dashed lines. H atoms not involved in the hydrogen bond interactions are omitted for clarity.

## supplementary crystallographic information

### Comment

The benzimidazole nucleus is the key building block for a variety of compounds that play crucial roles in the function of a number of biologically important molecules (Tanious *et al.*, 2004). Benzimidazole derivatives have shown different therapeutic properties such as antihypertensive (Kohara *et al.*, 1996) and anti-inflammatory (Mader *et al.*, 2008) activities. 2-(substitutedphenyl)benzimidazoles with various types of biological activities, such as antibacterial (Coburn *et al.*, 1987) and antiviral (Roth *et al.*, 1997), have been reported. Due to their importance, the crystal structure determination of the title compound was carried out and the results are presented here.

In the title molecule (Fig. 1), the imidazole ring is essentially planar with a maximum deviation of 0.004 (2) Å for atom C13. The dihedral angle between the imidazole ring (N1/N2/C13/C7–C8) and the benzene ring (C1–C6) is 66.33 (13)° . In the crystal structure (Fig. 2), the molecules are connected by weak C21—H21C···O1 (Table 1) hydrogen bonds, forming one-dimensional chains along the *b* axis.

### Experimental

Ethyl-3-amino-4-(*sec*-butylamino)benzoate (200 mg, 0.84 mmol) and the sodium metabisulfite adduct of 4-methoxy benzaldehyde (406 mg, 1.68 mmol) were dissolved in DMF. The reaction mixture was irradiated under microwave conditions at 403 K for 2 minutes. After completion of the reaction, the reaction mixture was diluted in EtOAc (20 ml) and washed with H_2_O (20 ml). The organic layer was collected, dried over Na_2_SO_4_ and then evaporated *in vacuo* to yield the crude product. The product was recrystallized from hot EtOAc to afford the title compound as colorless crystals.

### Refinement

All hydrogen atoms were positioned geometrically [C–H = 0.93–0.98 Å] and were refined using a riding model, with *U*_iso_(H) = 1.2 or 1.5 *U*_eq_(C). A rotating group model was applied to the methyl groups.

### Crystal data

**Table:**

C_21_H_24_N_2_O_3_	*F*(000) = 752
*M**_r_* = 352.42	*D*_x_ = 1.212 Mg m^−^^3^
Monoclinic, *P*2_1_/*c*	Mo *K*α radiation, λ = 0.71073 Å
Hall symbol: -P 2ybc	Cell parameters from 1865 reflections
*a* = 10.5815 (3) Å	θ = 2.6–19.8°
*b* = 12.1079 (3) Å	µ = 0.08 mm^−^^1^
*c* = 15.1050 (3) Å	*T* = 296 K
β = 93.678 (2)°	Plate, colourless
*V* = 1931.26 (8) Å^3^	0.40 × 0.31 × 0.07 mm
*Z* = 4	

### Data collection

**Table:**

Bruker SMART APEXII CCD area-detector diffractometer	4437 independent reflections
Radiation source: fine-focus sealed tube	2266 reflections with *I* > 2s(*I*)
graphite	*R*_int_ = 0.065
φ and ω scans	θ_max_ = 27.5°, θ_min_ = 1.9°
Absorption correction: multi-scan (*SADABS*; Bruker, 2009)	*h* = −13→13
*T*_min_ = 0.968, *T*_max_ = 0.994	*k* = −15→15
19216 measured reflections	*l* = −19→19

### Refinement

**Table:**

Refinement on *F*^2^	Primary atom site location: structure-invariant direct methods
Least-squares matrix: full	Secondary atom site location: difference Fourier map
*R*[*F*^2^ > 2σ(*F*^2^)] = 0.066	Hydrogen site location: inferred from neighbouring sites
*wR*(*F*^2^) = 0.150	H-atom parameters constrained
*S* = 1.04	*w* = 1/[σ^2^(*F*_o_^2^) + (0.0461*P*)^2^ + 0.4985*P*] where *P* = (*F*_o_^2^ + 2*F*_c_^2^)/3
4437 reflections	(Δ/σ)_max_ < 0.001
235 parameters	Δρ_max_ = 0.13 e Å^−^^3^
0 restraints	Δρ_min_ = −0.18 e Å^−^^3^

### Special details

**Table:**

Geometry. All esds (except the esd in the dihedral angle between two l.s. planes) are estimated using the full covariance matrix. The cell esds are taken into account individually in the estimation of esds in distances, angles and torsion angles; correlations between esds in cell parameters are only used when they are defined by crystal symmetry. An approximate (isotropic) treatment of cell esds is used for estimating esds involving l.s. planes.
Refinement. Refinement of F^2^ against ALL reflections. The weighted R-factor wR and goodness of fit S are based on F^2^, conventional R-factors R are based on F, with F set to zero for negative F^2^. The threshold expression of F^2^ > 2sigma(F^2^) is used only for calculating R-factors(gt) etc. and is not relevant to the choice of reflections for refinement. R-factors based on F^2^ are statistically about twice as large as those based on F, and R- factors based on ALL data will be even larger.

### Fractional atomic coordinates and isotropic or equivalent isotropic displacement parameters (Å^2^)

**Table:**

	*x*	*y*	*z*	*U*_iso_*/*U*_eq_	
O1	0.50185 (18)	0.95365 (16)	0.17576 (12)	0.0728 (6)	
O2	0.34173 (18)	1.00924 (15)	0.08352 (13)	0.0735 (6)	
O3	−0.47540 (19)	0.38119 (16)	0.08035 (13)	0.0761 (6)	
N1	0.07745 (19)	0.58199 (16)	0.20122 (13)	0.0503 (5)	
N2	0.00579 (19)	0.70718 (16)	0.10000 (13)	0.0532 (5)	
C1	−0.2515 (2)	0.5992 (2)	0.15122 (17)	0.0587 (7)	
H1A	−0.2505	0.6692	0.1767	0.070*	
C2	−0.3657 (3)	0.5442 (2)	0.13763 (16)	0.0594 (7)	
H2A	−0.4402	0.5765	0.1543	0.071*	
C3	−0.3678 (3)	0.4410 (2)	0.09909 (16)	0.0570 (7)	
C4	−0.2565 (3)	0.3940 (2)	0.07547 (19)	0.0696 (8)	
H4A	−0.2578	0.3243	0.0495	0.084*	
C5	−0.1438 (3)	0.4490 (2)	0.08990 (18)	0.0651 (8)	
H5A	−0.0693	0.4159	0.0739	0.078*	
C6	−0.1391 (2)	0.5534 (2)	0.12809 (15)	0.0491 (6)	
C7	−0.0199 (2)	0.6160 (2)	0.14217 (16)	0.0481 (6)	
C8	0.1732 (2)	0.65916 (19)	0.19625 (15)	0.0478 (6)	
C9	0.2938 (2)	0.6692 (2)	0.23777 (17)	0.0598 (7)	
H9A	0.3260	0.6164	0.2779	0.072*	
C10	0.3633 (2)	0.7602 (2)	0.21706 (17)	0.0563 (7)	
H10A	0.4438	0.7693	0.2445	0.068*	
C11	0.3174 (2)	0.8402 (2)	0.15602 (15)	0.0485 (6)	
C12	0.1987 (2)	0.8284 (2)	0.11322 (16)	0.0509 (6)	
H12A	0.1677	0.8803	0.0720	0.061*	
C13	0.1265 (2)	0.73630 (19)	0.13359 (15)	0.0452 (6)	
C14	0.3975 (3)	0.9378 (2)	0.14099 (17)	0.0548 (7)	
C15	0.4123 (3)	1.1098 (2)	0.0663 (2)	0.0797 (9)	
H15A	0.4977	1.0918	0.0515	0.096*	
H15B	0.4170	1.1571	0.1183	0.096*	
C16	0.3432 (3)	1.1663 (3)	−0.0091 (2)	0.0928 (10)	
H16A	0.3872	1.2328	−0.0229	0.139*	
H16B	0.2592	1.1843	0.0067	0.139*	
H16C	0.3385	1.1184	−0.0598	0.139*	
C17	0.0678 (3)	0.4949 (2)	0.26963 (17)	0.0600 (7)	
H17A	−0.0174	0.4634	0.2602	0.072*	
C18	0.0755 (3)	0.5434 (3)	0.36209 (18)	0.0758 (9)	
H18A	0.0617	0.4847	0.4041	0.091*	
H18B	0.1605	0.5715	0.3751	0.091*	
C19	−0.0167 (3)	0.6342 (3)	0.3763 (2)	0.0972 (11)	
H19A	−0.0046	0.6609	0.4361	0.146*	
H19B	−0.1015	0.6067	0.3662	0.146*	
H19C	−0.0033	0.6934	0.3357	0.146*	
C20	0.1589 (3)	0.4015 (2)	0.2570 (2)	0.0830 (10)	
H20A	0.1475	0.3744	0.1973	0.124*	
H20B	0.1432	0.3430	0.2977	0.124*	
H20C	0.2442	0.4276	0.2678	0.124*	
C21	−0.5922 (3)	0.4267 (3)	0.1060 (2)	0.0828 (10)	
H21A	−0.6601	0.3767	0.0894	0.124*	
H21B	−0.6072	0.4962	0.0766	0.124*	
H21C	−0.5877	0.4376	0.1690	0.124*	

### Atomic displacement parameters (Å^2^)

**Table:**

	*U*^11^	*U*^22^	*U*^33^	*U*^12^	*U*^13^	*U*^23^
O1	0.0634 (13)	0.0789 (14)	0.0747 (13)	−0.0233 (11)	−0.0070 (11)	0.0058 (10)
O2	0.0746 (13)	0.0553 (12)	0.0884 (14)	−0.0213 (10)	−0.0109 (11)	0.0173 (10)
O3	0.0636 (13)	0.0762 (14)	0.0884 (14)	−0.0216 (11)	0.0039 (10)	−0.0165 (11)
N1	0.0537 (13)	0.0410 (12)	0.0558 (12)	−0.0019 (10)	−0.0002 (10)	0.0107 (9)
N2	0.0560 (14)	0.0466 (12)	0.0557 (13)	−0.0065 (10)	−0.0052 (10)	0.0079 (10)
C1	0.0655 (18)	0.0428 (15)	0.0685 (18)	−0.0067 (14)	0.0085 (14)	−0.0055 (12)
C2	0.0556 (17)	0.0565 (17)	0.0668 (17)	−0.0023 (14)	0.0091 (13)	−0.0005 (14)
C3	0.0616 (18)	0.0545 (17)	0.0547 (15)	−0.0136 (15)	0.0015 (13)	−0.0006 (13)
C4	0.0677 (19)	0.0530 (17)	0.088 (2)	−0.0092 (16)	0.0052 (16)	−0.0216 (15)
C5	0.0599 (18)	0.0529 (17)	0.083 (2)	−0.0005 (15)	0.0062 (15)	−0.0119 (15)
C6	0.0534 (16)	0.0412 (14)	0.0524 (14)	−0.0027 (13)	0.0010 (12)	0.0048 (12)
C7	0.0520 (15)	0.0425 (14)	0.0498 (14)	−0.0003 (12)	0.0039 (12)	0.0028 (11)
C8	0.0496 (15)	0.0411 (14)	0.0527 (14)	−0.0007 (12)	0.0032 (12)	0.0026 (11)
C9	0.0554 (17)	0.0533 (17)	0.0699 (18)	0.0026 (14)	−0.0035 (14)	0.0114 (13)
C10	0.0494 (16)	0.0539 (17)	0.0643 (17)	−0.0004 (13)	−0.0050 (13)	0.0020 (13)
C11	0.0500 (15)	0.0440 (14)	0.0516 (14)	−0.0031 (12)	0.0050 (12)	−0.0023 (11)
C12	0.0575 (16)	0.0440 (15)	0.0506 (14)	−0.0013 (13)	−0.0005 (12)	0.0055 (11)
C13	0.0492 (15)	0.0399 (14)	0.0462 (14)	0.0016 (12)	0.0002 (11)	0.0005 (11)
C14	0.0575 (17)	0.0539 (17)	0.0531 (16)	−0.0077 (14)	0.0042 (14)	−0.0047 (13)
C15	0.092 (2)	0.0582 (19)	0.087 (2)	−0.0277 (17)	−0.0076 (18)	0.0144 (16)
C16	0.111 (3)	0.072 (2)	0.095 (2)	−0.022 (2)	0.003 (2)	0.0107 (19)
C17	0.0638 (17)	0.0486 (15)	0.0672 (18)	−0.0039 (14)	0.0018 (14)	0.0196 (13)
C18	0.081 (2)	0.083 (2)	0.0640 (19)	−0.0050 (18)	0.0050 (16)	0.0213 (16)
C19	0.108 (3)	0.110 (3)	0.074 (2)	0.020 (2)	0.0124 (19)	−0.008 (2)
C20	0.079 (2)	0.0545 (18)	0.116 (3)	0.0092 (17)	0.0115 (19)	0.0259 (17)
C21	0.063 (2)	0.107 (3)	0.078 (2)	−0.0206 (19)	0.0052 (16)	−0.0121 (19)

### Geometric parameters (Å, °)

**Table:**

O1—C14	1.208 (3)	C10—H10A	0.9300
O2—C14	1.335 (3)	C11—C12	1.383 (3)
O2—C15	1.461 (3)	C11—C14	1.481 (3)
O3—C3	1.363 (3)	C12—C13	1.396 (3)
O3—C21	1.429 (3)	C12—H12A	0.9300
N1—C7	1.381 (3)	C15—C16	1.480 (4)
N1—C8	1.384 (3)	C15—H15A	0.9700
N1—C17	1.485 (3)	C15—H15B	0.9700
N2—C7	1.312 (3)	C16—H16A	0.9600
N2—C13	1.389 (3)	C16—H16B	0.9600
C1—C6	1.377 (3)	C16—H16C	0.9600
C1—C2	1.384 (3)	C17—C20	1.507 (4)
C1—H1A	0.9300	C17—C18	1.512 (4)
C2—C3	1.379 (4)	C17—H17A	0.9800
C2—H2A	0.9300	C18—C19	1.495 (4)
C3—C4	1.375 (4)	C18—H18A	0.9700
C4—C5	1.372 (4)	C18—H18B	0.9700
C4—H4A	0.9300	C19—H19A	0.9600
C5—C6	1.389 (3)	C19—H19B	0.9600
C5—H5A	0.9300	C19—H19C	0.9600
C6—C7	1.475 (3)	C20—H20A	0.9600
C8—C9	1.391 (3)	C20—H20B	0.9600
C8—C13	1.397 (3)	C20—H20C	0.9600
C9—C10	1.373 (3)	C21—H21A	0.9600
C9—H9A	0.9300	C21—H21B	0.9600
C10—C11	1.402 (3)	C21—H21C	0.9600
			
C14—O2—C15	116.5 (2)	O1—C14—O2	122.2 (2)
C3—O3—C21	117.7 (2)	O1—C14—C11	125.2 (3)
C7—N1—C8	106.39 (19)	O2—C14—C11	112.6 (2)
C7—N1—C17	125.4 (2)	O2—C15—C16	106.8 (2)
C8—N1—C17	126.9 (2)	O2—C15—H15A	110.4
C7—N2—C13	104.5 (2)	C16—C15—H15A	110.4
C6—C1—C2	121.9 (2)	O2—C15—H15B	110.4
C6—C1—H1A	119.0	C16—C15—H15B	110.4
C2—C1—H1A	119.0	H15A—C15—H15B	108.6
C3—C2—C1	119.3 (2)	C15—C16—H16A	109.5
C3—C2—H2A	120.3	C15—C16—H16B	109.5
C1—C2—H2A	120.3	H16A—C16—H16B	109.5
O3—C3—C4	116.4 (2)	C15—C16—H16C	109.5
O3—C3—C2	124.0 (3)	H16A—C16—H16C	109.5
C4—C3—C2	119.6 (3)	H16B—C16—H16C	109.5
C5—C4—C3	120.5 (3)	N1—C17—C20	111.8 (2)
C5—C4—H4A	119.7	N1—C17—C18	111.4 (2)
C3—C4—H4A	119.7	C20—C17—C18	114.3 (2)
C4—C5—C6	121.0 (3)	N1—C17—H17A	106.2
C4—C5—H5A	119.5	C20—C17—H17A	106.2
C6—C5—H5A	119.5	C18—C17—H17A	106.2
C1—C6—C5	117.6 (2)	C19—C18—C17	114.9 (2)
C1—C6—C7	120.1 (2)	C19—C18—H18A	108.5
C5—C6—C7	122.3 (2)	C17—C18—H18A	108.5
N2—C7—N1	113.3 (2)	C19—C18—H18B	108.5
N2—C7—C6	124.3 (2)	C17—C18—H18B	108.5
N1—C7—C6	122.4 (2)	H18A—C18—H18B	107.5
N1—C8—C9	133.5 (2)	C18—C19—H19A	109.5
N1—C8—C13	105.1 (2)	C18—C19—H19B	109.5
C9—C8—C13	121.4 (2)	H19A—C19—H19B	109.5
C10—C9—C8	117.2 (2)	C18—C19—H19C	109.5
C10—C9—H9A	121.4	H19A—C19—H19C	109.5
C8—C9—H9A	121.4	H19B—C19—H19C	109.5
C9—C10—C11	122.4 (2)	C17—C20—H20A	109.5
C9—C10—H10A	118.8	C17—C20—H20B	109.5
C11—C10—H10A	118.8	H20A—C20—H20B	109.5
C12—C11—C10	120.2 (2)	C17—C20—H20C	109.5
C12—C11—C14	121.5 (2)	H20A—C20—H20C	109.5
C10—C11—C14	118.3 (2)	H20B—C20—H20C	109.5
C11—C12—C13	118.2 (2)	O3—C21—H21A	109.5
C11—C12—H12A	120.9	O3—C21—H21B	109.5
C13—C12—H12A	120.9	H21A—C21—H21B	109.5
N2—C13—C12	128.7 (2)	O3—C21—H21C	109.5
N2—C13—C8	110.8 (2)	H21A—C21—H21C	109.5
C12—C13—C8	120.5 (2)	H21B—C21—H21C	109.5
			
C6—C1—C2—C3	−0.6 (4)	C13—C8—C9—C10	2.4 (4)
C21—O3—C3—C4	178.6 (2)	C8—C9—C10—C11	−0.8 (4)
C21—O3—C3—C2	−2.9 (4)	C9—C10—C11—C12	−0.8 (4)
C1—C2—C3—O3	−178.0 (2)	C9—C10—C11—C14	177.5 (2)
C1—C2—C3—C4	0.5 (4)	C10—C11—C12—C13	0.9 (3)
O3—C3—C4—C5	178.6 (3)	C14—C11—C12—C13	−177.3 (2)
C2—C3—C4—C5	0.0 (4)	C7—N2—C13—C12	−178.3 (2)
C3—C4—C5—C6	−0.4 (4)	C7—N2—C13—C8	0.8 (3)
C2—C1—C6—C5	0.2 (4)	C11—C12—C13—N2	179.7 (2)
C2—C1—C6—C7	178.8 (2)	C11—C12—C13—C8	0.6 (3)
C4—C5—C6—C1	0.3 (4)	N1—C8—C13—N2	−0.6 (3)
C4—C5—C6—C7	−178.3 (3)	C9—C8—C13—N2	178.5 (2)
C13—N2—C7—N1	−0.7 (3)	N1—C8—C13—C12	178.6 (2)
C13—N2—C7—C6	−179.7 (2)	C9—C8—C13—C12	−2.3 (4)
C8—N1—C7—N2	0.3 (3)	C15—O2—C14—O1	−1.6 (4)
C17—N1—C7—N2	168.1 (2)	C15—O2—C14—C11	178.4 (2)
C8—N1—C7—C6	179.4 (2)	C12—C11—C14—O1	−179.9 (2)
C17—N1—C7—C6	−12.9 (4)	C10—C11—C14—O1	1.9 (4)
C1—C6—C7—N2	−66.1 (3)	C12—C11—C14—O2	0.1 (3)
C5—C6—C7—N2	112.4 (3)	C10—C11—C14—O2	−178.1 (2)
C1—C6—C7—N1	114.9 (3)	C14—O2—C15—C16	170.8 (2)
C5—C6—C7—N1	−66.5 (3)	C7—N1—C17—C20	120.4 (3)
C7—N1—C8—C9	−178.8 (3)	C8—N1—C17—C20	−74.4 (3)
C17—N1—C8—C9	13.8 (4)	C7—N1—C17—C18	−110.3 (3)
C7—N1—C8—C13	0.2 (2)	C8—N1—C17—C18	54.9 (3)
C17—N1—C8—C13	−167.3 (2)	N1—C17—C18—C19	53.6 (3)
N1—C8—C9—C10	−178.9 (2)	C20—C17—C18—C19	−178.5 (3)

### Hydrogen-bond geometry (Å, °)

**Table:**

*D*—H···*A*	*D*—H	H···*A*	*D*···*A*	*D*—H···*A*
C21—H21C···O1^i^	0.96	2.47	3.389 (4)	161

Symmetry codes: (i) −*x*, *y*−1/2, −*z*+1/2.
